# Evaluating the concept of three-dimensional printing guided endodontics in the dog

**DOI:** 10.3389/fvets.2024.1481612

**Published:** 2024-11-21

**Authors:** Jiahui Peng, Jie Yang, Ruiyu Liu, Haifeng Liu, Zhijun Zhong, Guangneng Peng, Kun Zhang, Chengli Zheng, Ming Zhang, Ziyao Zhou

**Affiliations:** ^1^College of Veterinary Medicine, Sichuan Agricultural University, Chengdu, China; ^2^Sichuan Institute of Musk Deer Breeding, Chengdu, China; ^3^College of Animal Science and Technology, Sichuan Agricultural University, Chengdu, China

**Keywords:** guided endodontics, finite element analysis, 3D printing, root canal, computer-aided design

## Abstract

**Introduction:**

Guided endodontics represents an effective method for achieving safe and reliable endodontic surgery in human medicine. However, it is rarely employed in small animal dentistry. This study employed finite element analysis and three-dimensional (3D) printing techniques to explore the feasibility of guided endodontics in Beagle mandibular teeth.

**Methods:**

The methodology included the processing of Computed Tomography (CT) data, the creation of mathematical and 3D printing templates of the root canal pathways, and the evaluation of dog 3D printing guided endodontics compared with classic root canal method using radiograph.

**Results:**

In this experiment, the coordinates of the central point of pulp crown and apex point for each tooth were recorded. Based on the extension line of the central point of dental root canal orifice and the apex point, guided endodontic templates were designed on each root canal of 20 teeth in the Beagle mandible. Among them, the average relative deviation of guided endodontics and classic root canal method was 4.28%  ±  2.75%, and the mean angular deviation was 1.90  ±  0.25°.

**Discussion:**

Our research indicated that dog 3D printing guided endodontics has accurate position, direction, and length, which may assist veterinary dentistry in root canal treatment in small animals.

## 1 Introduction

Enamel in dogs exhibits a particularly thin component in thickness (even <0.1 mm), making it susceptible to damage during functional activities, leading to lesions in the pulp (1). Consequently, pulp and periapical diseases represent the most prevalent dental conditions encountered in veterinary practice (2). As such, root canal treatment is the primary and most effective intervention for addressing pulp and periapical lesions both in human and veterinary medicine (3). This therapeutic approach effectively alleviates symptoms associated with pulpitis, periapical inflammation, and pulp necrosis (4). Recently, guided endodontics has been proven to be an effective method for achieving favorable outcomes in human patients (5). However, its application in small animal dentistry remains relatively unknown, primarily due to the limited access of veterinary dentistry to sophisticated specialized equipment (6). With the development of guided dental implantation utilizing computed tomography (CT) scans and three-dimensional (3D) finite element analysis (FEA), a viable solution for precise access in teeth with complex dental anatomy in veterinary dentistry may be available (7–9). It has the potential to achieve predictable and safe outcomes by providing clinicians with virtual visualization of the drilling position and angle during the planning stage, facilitating necessary adjustments and enhancing procedural predictability (10).

A precisely guided endodontic approach requires not only advanced imaging equipment, but also suitable image processing software. The study employed several FEA operating software to facilitate this process. The first software used was Mimics Medical (11), which is specially designed for CT, magnetic resonance imaging and other medical image processing. Its primary function is to extract valuable information from medical images and apply it to both clinical practice and research endeavors. 3D digital softwares, Geomagic Wrap (12) and SOLIDWORKS (13), were also necessary in create and computer-aided design (CAD) complex 3D models, including the assembly, analysis, and simulation. In recent decades, 3D printing technology has emerged as a significant tool in the field of dentistry. It can transform digital imaging data into highly accurate 3D models enables the precise replication of anatomical structures and further make the virtual visualization into real-time guidance (14). Therefore, it has the potential to support the application of guided endodontics in the veterinary dentistry. The integration of the specialized software, in conjunction with advanced imaging and 3D printing technology enables the precise planning and execution of guided endodontics.

In theory, guided endodontics simulated by FEA should have a very accuracy for the surgical path. However, the 3D printing technology and the operational experiences current in veterinary dentistry remains relatively unknown, may make deviations in the clinical practices. Here, the objective of this study is to investigate the accuracy of guided endodontics of Beagle mandibular teeth using FEA and 3D-printed templates, compared with the classic root canal method. Our study involves simulating the treatment of root canal treatment by the processing of CT data, the creation of mathematical, 3D printing of the guided endodontics, and the evaluation of the root canal pathways by radiograph, which presents a novel approach to addressing the challenges faced in veterinary endodontic treatments in small animals.

## 2 Materials and methods

### 2.1 Establishment of a computer model of dog mandible

**Figure 1:**
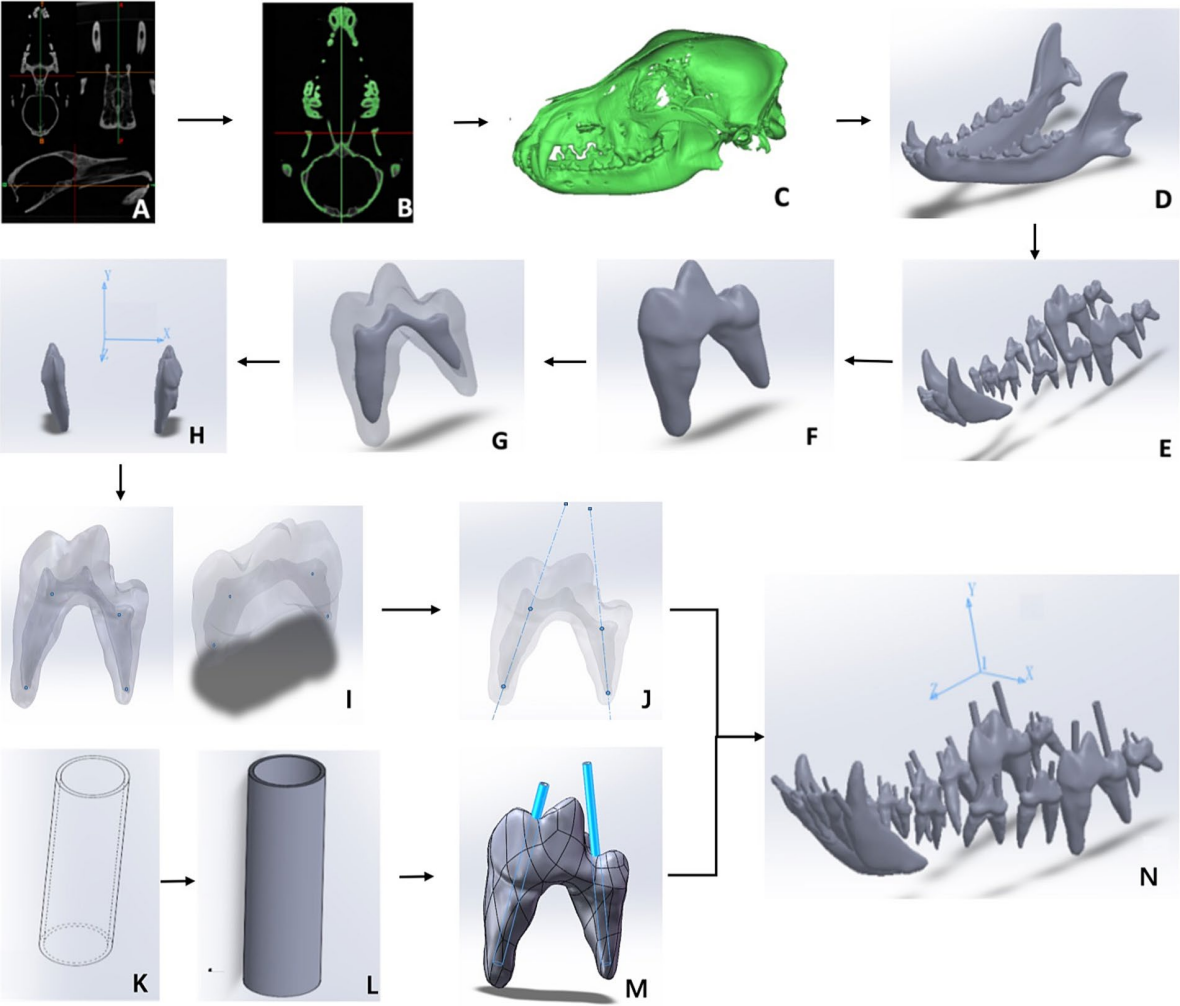
Scheme of 3D model and finite element analysis. **(A)** The Beagle CT scan data. **(B)** Head skull mask image generated. **(C)** Preliminary 3D skull model extracted. **(D)** Elaborated 3D model of dog mandible. **(E)** Teeth Extracted. **(F)** Single tooth extracted. **(G)** Reconstruction of dental pulp. **(H)** 3D positions of each tooth with xyz axes. **(I)** Central point of dental root canal orifice and the apex point generated. **(J)** Virtual root canal lines generated. **(K)** A virtual guidance of the root canal. **(L)** A precisely guidance of the root canal. **(M)** Assembly of the guidance into the dental pulp. **(N)** The assembled mandible tooth and guidance with 3D coordinate system.

A CT scan was conducted on a healthy adult Beagle in accordance with the manufacturer’s instructions (uCT503e, 40-slice detector, 40 slices, voltage 120 kV, current 250 mA, reconstruction matrix 512 × 512, slice thickness 1 mm, bone window reconstruction algorithm with window width of 2,600 HU and window position of 800 HU, soft tissue window reconstruction algorithm with window width of 300 HU and window position of 40 HU, Chengdu Ultrasound Imaging Center Co., Ltd., China). The dog was without any fractures, osteoporosis, tumors, or other head diseases. The CT images (Figure 1A) were imported into Mimics Medical (Materialise Co., Ltd. Belgium, Version 21.0.406) in DICOM format for processing. First, the “New Mask” function was used to create a 3D mesh model of the dog mandible (Figure 1B). Secondly, the “Edit Mask” function was used to edit the mask area, deleting the maxilla and skull and separating the bone fragments of the mandibular canines, premolars, and molars that were connected to the maxilla. The “Region growing” function was used to generate a structure separate from the other regions, which resulted in a coarse sample of the dog mandible model (Figure 1C). Cortical bone and cancellous bone were separated using the “Erosion” function. The stereolithography file was then imported into Geomagic Wrap (Geomagic Co., Ltd. United States, version 2021.0.0.3008). The surface was smoothed using the “Feature Removal” and “Hole Filling” functions. The “Precise Surface” function was next employed to accurately fit the surface, allowing for selective editing and removal of noise, thereby generating a 3D model of the mandibular bone. Finally, the mandibular graphics was exported in STP format and import it into SOLIDWORKS (Dassault Systemes S.A Co., Ltd. France, version 29.0.0.5028) for materialization (Figure 1D).

### 2.2 Extraction and isolation of mandibular teeth

In SOLIDWORKS software, 20 teeth in the Beagle mandibular bone were located and virtual extracted (Figure 1E). Subsequently, “Region Growing” function was used to eliminate non-continuous noise. The entire tooth was reconstructed using “3D Editing” and “Boolean operation” to remove artifact parts for each individual tooth, which makes the tooth converted into a fine surface polygon (Figure 1F) (15). The pulp cavity and dentin were reconstructed separately (Figure 1G) (16). Next, the “Coordinate System” function was used in the SOLIDWORKS software to generate a 3D coordinate system comprising the XYZ axes, exported in STL format (Figure 1H).

### 2.3 Establishment of a model for root canals

In SOLIDWORKS software, the anatomical center of the skull was taken as the origin coordinates (0, 0, 0). The “reference geometry” function was employed to identify the coordinates of the central point of dental root canal orifice (x_1_, y_1_, z_1_) and the apex point (x_2_, y_2_, z_2_) for each tooth (Figure 1I). Theoretically, there exists a straight line between two points in 3D space (17). The “Curve Through XYZ Points” function was performed to output the root canal linear coordinates according to the two points for each tooth (Figure 1J).

Based on the extension line of the root canal and the tooth surface, a root canal guidance sleeve was built outwards with a thickness of 1 mm. With the diameter of the maximum root canal diameter, the proportional size guidance models were next stretched into a 3D hollow cylinder (Figures 1K,L). The guidance was extended up to the incisal edge to allow for straight-line access (Figure 1M). In order to ensure the accuracy of the positions of each tooth, we also performed the entire skull (Figure 1N). The resulting model was exported as a an “STL” file.

### 2.4 *In vitro* root canal experiment

The *in vitro* root canal experiment was conducted on separate copies of the same Beagle dog. A total of 30 root canals from 20 lower jaw teeth (including 6 incisors, 2 canines, 8 premolars and 4 molars) of Beagle were subjected to root canal treatment. Initially, the template of each tooth was printed according to the manufactory instruction (SLA550Li, material: photosensitive resin, Ultrust Imaging (Chengdu) Center Co., Ltd., China). Two separate copies of the same model were produced for the purposes of guided endodontic and classic method treatments, respectively. The first mandibular model was used for guided endodontic treatment, and the 3D-printed guide root canal template was placed on the tooth. The correct and reproducible fitting of template and the corresponding tooth was firstly checked. The cavity was extended up to the incisal edge to allow for straight-line access, parallel to the long axis of the tooth for the drill, establishing a preparatory path for entry into the dental pulp and determining the working length of the root canal. The second mandibular model was used for root canal treatment using the classic method. The root canal path of the classic method was implemented including pre-operative radiographs and open pulp access by a certified small animal dentist of Chinese Veterinary Medical Association. The lengths of every root canal by two methods were measured from the central of dental root canal orifice to the apex. The angular deviation of the two methods were evaluated to verify the accuracy of the guided endodontic pathway compared to the classic method.

### 2.5 Statistical analysis

Statistical analysis was conducted on the measured root canal lengths and the angular deviations of the two methods. The statistical comparison of data distribution was tested by Student’s *t*-test using SPSS v27 (IBM, New York, United States). *p* < 0.05 was considered as statistically significant.

## 3 Results

### 3.1 Computer-aided designed root canal guided lines

In the dental root canal digital assembly, the diameter of the coronal pulp of the mandible tooth was measured and recorded in Supplementary Table S1. In accordance with the spatial coordinate system of the xyz axis, a total of 30 guided endodontic templates were fabricated on 20 teeth in the mandible. Thirty coordinates of the central point of dental root canal orifice (x1, y1, z1) and the apex point (x2, y2, z2) for each tooth were corroborated (Table 1). The left first molar (309) as an example illustrated the results of the root canal digital assembly of a 3D stereo mandibular model.

**Table 1:** Coordinates of the central point of dental root canal orifice (x_1_, y_1_, z_1_) and the apex point (x_2_, y_2_, z_2_) for each tooth.

Tooth number	Root position	Roots of teeth (x, y, z)	Tooth crown (x, y, z)	Tooth number	Root position	Roots of teeth (x, y, z)	Tooth crown (x, y, z)
301	/	(0.85, −7.66, 59.98)	(−0.28, −16.17, 56.47)	401	/	(−1.14, −8.14, 60.61)	(−1.03, −17.72, 57.77)
302	/	(3.04, −6.86, 58.02)	(1.06, −20.25, 55.87)	402	/	(−3.56, −7.36, 59.90)	(−1.80, −19.19, 54.26)
303	/	(6.91, −9.22, 56.85)	(1.82, −22.14, 49.39)	403	/	(−7.36, −9.62, 58.43)	(−2.33,-22.97, 49.78)
304	/	(9.46, −12.75, 52.87)	(3.43, −33.66, 30.15)	404	/	(−9.11, −13.32, 52.32)	(−3.54, −33.14, 29.93)
305	/	(8.29, −18.02, 35.51)	(6.67, −25.01, 36.56)	405	/	(−8.47, −19.14, 34.91)	(−6.36, −26.03, 36.83)
306	Mesial	(8.54, −20.35, 32.23)	(6.83, −27.10, 33.44)	406	Mesial	(−8.86, −21.11, 32.16)	(−6.33, −27.78, 33.84)
	Distal	(9.44, −23.15, 26.73)	(6.98, −29.84, 27.68)		Distal	(−9.26, −23.26, 25.17)	(−7.44, −28.11, 28.77)
307	Mesial	(12.38, −25.18, 16.30)	(9.04, −32.15, 19.61)	407	Mesial	(−13.12, −25.30, 14.69)	(−9.39, −33.15, 18.40)
	Distal	(13.15, −25.96, 12.83)	(11.53, −34.22, 11.27)		Distal	(−14.63, −26.04, 10.47)	(−12.07, −33.89, 10.07)
308	Mesial	(16.37, −27.65, 2.05)	(12.30, −37.04, 6.79)	408	Mesial	(−16.24, −27.66, −1.69)	(−12.70, −37.06, 5.59)
	Distal	(18.36, −27.93, −2.30)	(13.89, −38.19, −2.79)		Distal	(−18.85, −26.68, −2.75)	(−14.82, −37.30, −4.00)
309	Mesial	(18.66, −25.52, −10.92)	(15.21, −43.26, −9.75)	409	Mesial	(−18.56, −26.47, −12.91)	(−16.03, −43.84, −10.24)
	Distal	(18.86, −28.54, −20.68)	(19.51, −40.51, −23.97)		Distal	(−19.84, −29.40, −20.91)	(−18.34, −41.64, −24.63)
310	Mesial	(19.25, −27.70, −28.22)	(20.46, −35.61, −27.52)	410	Mesial	(−19.03, −28.50, −30.04)	(−20.36, −36.62, −27.71)
	Distal	(19.85, −28.11, −32.64)	(20.89, −33.91, −38.83)		Distal	(−20.06, −28.65, −33.59)	(−20.88, −34.22, −38.35)

The anatomical center of the skull was taken as the origin coordinates (0, 0, 0).

The diameters of the two dental root canals of the Beagle’s left first molar (309) were 5.91 mm and 5.05 mm, respectively. The coordinates of the mesial root of the first molar were measured in 3D space were (18.66, −25.52, −10.92) and (15.21, −43.26, −9.75), respectively. The coordinates of distal root were (18.86, −28.54, −20.68) and (19.51, −40.51, −23.97), respectively. There, root canal guided lines could be draw in the teeth according to the points.

### 3.2 3D-printed mandibular and guided endodontic templates

**Figure 2:**
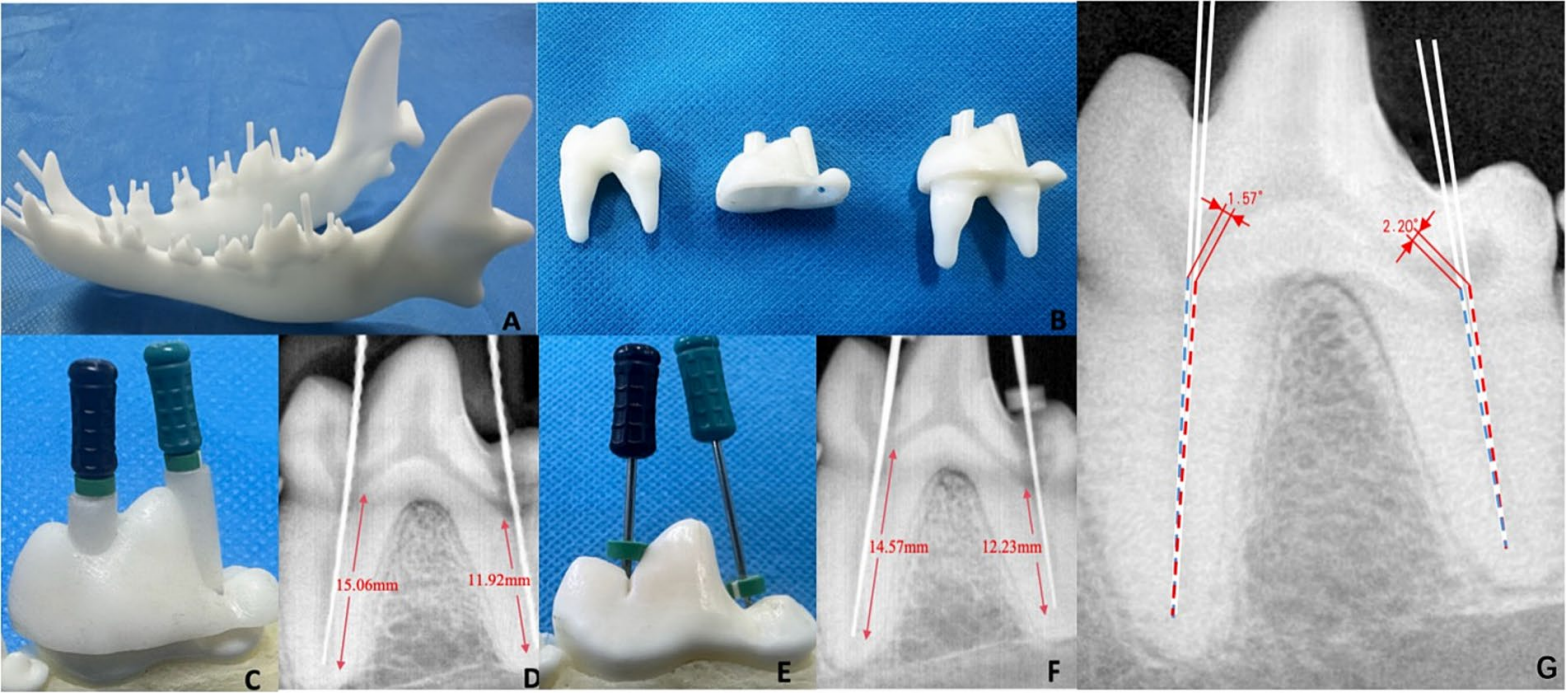
Evaluation of dog 3D printing guided endodontics. **(A)** 3D printed model of guided root canal in the mandible. **(B)** 3D printed root canal template and first molar tooth. **(C)** Root canal treatment using guided template. **(D)** Radiograph of 3D printing guided endodontics. **(E)** Root canal treatment using classic method. **(F)** Radiograph of classic root canal method. **(G)** The angular deviation measurement of the guided method (blue line) and the classical method (red line).

3D printing was performed through the established model, illustrated a precisely fitting of template and printed teeth (Figure 2A). Individual measurements of the length, width, and height of each tooth in the virtual extracted Beagle’s teeth were also made (Supplementary Table S2). Specifically, the first molar had a length of 20.43 mm, a width of 7.87 mm, and a height of 13.95 mm. The template size was 24.43 mm × 11.87 mm × 15.95 mm. This template was fully compatible with the first molar (Figure 2B). The linear pathway was introduced which is embedded in the pulp chamber (Figure 2C). The maximum diameter of the root canal sleeve of the first molar in the picture was essentially anastomosis to the square diameter of the coronal pulp. From a fit perspective, the guided endodontic template for the first molar might not cause the deviation of the orientation and angle after the insertion of root canal instruments (Figure 2D).

### 3.3 Comparison of 3D printing guided endodontics and classic root canal method

In the evaluation experiment, root canal treatment was performed on a total of 30 pulp chambers from 20 mandibular teeth in Beagle. Taking the first molar as an example, the radiographic examination revealed that the root canals was a direct line reached the apex without bent, damaged, or excessively long. The length of the mesial root canal was 15.06 mm, while the length of the distal root canal was 11.92 mm (Figure 2D). On the other hand, the length of the mesial root canal of the first molar using classic root canal method was 14.57 mm, while the distal root canal was 12.23 mm (Figures 2E,F). The angular deviation of root canal pathway between two methods were 1.57° (mesial root) and 2.20° (distal root), respectively (Figure 2G). For the clarification, to make the figure readable and measurable, the two virtual lines of Figure 2G were redraw by the operated root canal pathways of two methods in a new tooth of separate copies of the same Beagle dog. Of the 30 root canals, the relative deviation of the guided endodontics and classic methods were from 0.57% (304 root) to 10.71% (408 distal root), with the average deviation 4.28 ± 2.75%. The mean angular deviation was 1.90° ± 0.25°, with a range of 1.50° (306 distal root) to 2.33° (310 distal root) (Table 2).

**Table 2:** The length of each root canal measured with guided endodontics and classic method.

Tooth number	Root position	Guided endodontics/mm	Classic method/mm	Deviation rate	Angular deviation	Tooth number	Root position	Guided endodontics/mm	Classic method/mm	Deviation rate	Angular deviation
301	/	9.11	9.27	1.73%	2.34°	401	/	9.75	9.82	0.71%	1.65°
302	/	13.27	13.71	3.21%	2.12°	402	/	13.43	13.22	1.59%	1.89°
303	/	15.44	15.76	2.03%	1.97°	403	/	14.6	15.07	3.12%	2.02°
304	/	31.28	31.46	0.57%	1.56°	404	/	31.03	30.62	1.34%	1.76°
305	/	6.52	6.25	4.32	1.88°	405	/	6.85	6.33	8.12%	2.21°
306	Mesial	6.48	6.06	6.93%	1.75°	406	Mesial	6.98	7.12	1.97%	1.62°
	Distal	7.74	7.19	7.65%	1.50°		Distal	7.29	7.19	1.39%	2.07°
307	Mesial	8.08	8.41	3.92%	2.14°	407	Mesial	8.83	9.44	6.46%	2.12°
	Distal	7.95	8.56	7.13%	1.62°		Distal	7.78	8.09	3.83%	1.79°
308	Mesial	9.81	9.28	5.71%	1.72°	408	Mesial	10.67	10.38	2.79%	1.88°
	Distal	9.98	9.20	8.48%	1.85°		Distal	10.44	9.43	10.71%	1.59°
309	Mesial	15.06	14.57	3.36%	1.57°	409	Mesial	15.56	14.76	5.42%	1.82°
	Distal	11.92	12.23	2.53%	2.20°		Distal	12.27	12.88	4.74	2.06°
310	Mesial	8.23	8.03	2.49%	2.33°	410	Mesial	9.00	8.55	5.26%	2.13°
	Distal	6.44	6.55	1.68%	1.51°		Distal	8.04	7.37	9.09%	1.97°

Among each kind of teeth, the premolars had the most variation in the length, with 5.57% ± 2.72%. between guided endodontics and the classic method, significantly higher than incisors (2.07% ± 0.96%, *p* = 0.0170), but similar with the molars (4.32% ± 0.37%, *p* = 0.0515). However, there was no significant difference of the angular deviation between the teeth, with incisors 2.00° ± 0.23°, premolars 1.84° ± 0.23° and molars 1.95° ± 0.29°, respectively.

## 4 Discussion

Currently, root canal treatment is largely dependent on the expertise and technical proficiency of experienced dentists. There are several potential causes of failure in this surgical intervention, including: (1) Deviation in the access to the root canal treatment: incorrect angulation of pulp chamber access, incomplete exposure of pulp chamber or inadequate exposure of root canal orifices, perforation of the chamber floor, perforation of the root canal walls, missed canals, or misinterpretation of root canal anomalies for example C-shaped root canal configurations (18); (2) Deviation in root canal preparation: excessive enlargement of the root canal, formation of ledges, instrument separation, or incomplete cleaning of the root canal. For example, Lee et al. (19) conducted an evaluation of the outcomes of root canal treatment in 281 teeth across 204 dogs, with multirooted teeth having more complex root morphology being observed to have less favorable outcomes. Kuntsi-Vaattovaara et al. (20) assessed the results of root canal procedures in 127 cases and found that the failure rate increased due to root canal fracture, secondary tooth fracture and incorrect treatment protocols. In such instances, root canal treatment might become exceedingly challenging, often resulting in higher rates of treatment failure (21, 22). The heavy reliance on the individual expertise of dental practitioners is a significant limitation in the field of endodontics especially in small animal dentistry. Developing an aid approach for root canal treatment could help mitigate the risk of complications and improve the overall prognosis for patients. In order to minimize the risk of technical errors and shorten the treatment time, computer-aided designed methods were developed to minimally invasively locate the root canal, coined “guided endodontics” (23).

Laboratory and *in vitro* studies have reported a high level of accuracy when comparing the actual instrumented root canal pathway to the pre-planned approach using guided techniques (24, 25). Furthermore, these guided endodontic methods have demonstrated promising success rates when applied in clinical cases in human medicine (26). For instance, Jain et al. (27) conducted a study evaluating the use of conservative access cavity preparation on a 3D-printed tooth model consisting of 84 teeth and 138 root canals, found that the guided approach enabled high precision in accessing the root canal. Similarly, Chong et al. (28) performed conservative access cavity preparation on intact extracted human teeth using guided methods, achieving a success rate of 89%. Additionally, Torres et al. (29) used guided endodontic treatment reported an impressive accuracy rate of 93% in locating and instrumenting the root canals. Prior to initiating treatment, it is advantageous to simulate the entire process using computer technology. This methodology enables veterinary to formulate a feasible plan for actual clinical operations for best location and angulation, ultimately minimizing removal of precious tooth structure.

However, the application of guided endodontics in small animal dentistry remains unknown. Changes in tooth morphology, varying degrees of tooth wear and inconsistent pulp orientation are all key factors affecting root canal treatment, for instance, canine teeth typically possess a single pulp that is both narrower and longer, whereas the first molar contains two pulps with their respective roots oriented in different directions. Therefore, our study utilized FEA and 3D printing technology to perform and evaluate the efficacy of guided endodontics in dog mandibular teeth. Our results indicated that the guided endodontics was similar to the classic root canal method by a dentistry specialist. This guided endodontics relies on a series of specialized software that incorporates preoperative CT imaging data and FEA. Through there are various dental implantation design software, such as Pro/E (30), 3Shape (31), and DentalNavi (32), it seems to be some limitations to designing small animal root canal templates. For example, some of pulps have curved structures within the teeth which make root position varies, the specific implantation design software is hard to accurately determine the coordinates of root and crown in three-dimensional space. Additionally, the specific implantation design software may only include professional functions that relevant with implantation pathway. Therefore, it enable us to create templates with a comprehensive flow that closely with the characteristics of the pulps. Given the inherent complexity and uncertainty associated with clinical surgical procedures, the preoperative simulation of guided endodontics might be a complementary component for operation planning and execution.

The advantages of the guided endodontics include: (1) FEA, when integrated with patient-specific CT data, may provide highly accurate representations of the individual’s anatomical conditions and precise 3D information; (2) The capability to generate personalized surgical guide tools and customized implants may reduce the manufacturing product time and costs (33). (3) Preoperative planning can be significantly enhanced and thus increasing the success rate of surgical interventions. However, the series of FEA software for the guided endodontics required a high level of operational complexity, as well as a solid foundation in computer science. When measuring the length of the pulp chamber, it is necessary to strategically plan the insertion path for root canal based on specific crown-based measurement points. Compared to the classic root canal treatment, guided endodontics might help determining the most appropriate position for accessing the root canal, thus reducing the operational requirements. Additionally, some studies have reported the benefits of this minimally invasive, guided approach, including increased fracture resistance and maximum preservation of the natural tooth structure (34, 35). Compared to the classic root canal treatment, the biomechanical advantages of guided endodontics may preserve tooth structure and can contribute to improved postoperative patient recovery and healing (36). Currently, in the field of veterinary medicine, there is insufficient literature and research to demonstrate that the method of guided endodontics in the biomechanics and postoperative healing of animal teeth, which needed further verification.

In our guided endodontics experience, it is of paramount importance to ensure a precise fit between the constructed guided endodontic template and the root canal, which is critical to preventing the introduction of skewing direction during the clinical intervention (37). Therefore, prior to print the 3D guided endodontic template, it is strongly recommended to manually ensure fit the dimensions of coordinate points within the SOLIDWORKS software, thereby avoiding any mismatch between the template and root canal after the final assembly. Additionally, given the paramount importance of the appropriate cavity depth and direction in the clinical procedure, it is essential to thoroughly re-confirm the length and size of the digital simulated guided endodontics. In some cases, it may even be necessary to re-measure the numerical parameters of the root canal and perform a second positioning assembly to ensure a proper fit. However, there was inescapable error between the operation results and anticipatory plan of 3D model. In our results, the differences in root canal length of premolars were significantly higher than those of incisors, which may be the two root canals in some premolars shared a common open pulp access, which affects the difficulty of operation. However, there was no significant difference of the angular deviation between the teeth, with only 1.90° ± 0.25°, which may good suitable for clinical operation.

However, it is important to acknowledge the inherent limitations associated with this study. The study utilized Beagle dogs for the FEA, and the data parameters and guided endodontic templates may not be directly applicable to other dog breeds. Due to variations in body types, gender, and age, as well as changes in dental structure, the root canal templates generated by dogs of the same breed can differ significantly. Complex dental cases often come with increased operational demands, presenting a significant challenge to the skills and experience of the practitioner (38). Although, as a preliminary experiment, we did not evaluate 3D printing in complex root canal treatment such as C-shaped root canal, it is certainly a good application for guided endodontics (39). Additionally, this method has stringent requirements for the use of oral medical devices and often necessitates more time and resources to minimize the likelihood of surgical failure. On the other hand, the template method involves CT scanning, allowing for a comprehensive simulation of the entire surgical process within specialized software. While designing and printing templates may take some additional time, it can provide a reference idea for guiding root canal treatment, and also play a certain role in promoting the promotion of root canal treatment. Furthermore, most 3D printing materials are composed of a single resin material (40), which may not accurately replicate the complex structural and material properties of natural dentition. Guided endodontics with FEA represents a safe and directly approach, particularly considering the potential for further development in 3D printing technology (41). However, further clinical research may be required to comprehensively evaluate the clinical benefits of using emerging guided techniques.

## Data Availability

The original contributions presented in the study are included in the article/Supplementary material, further inquiries can be directed to the corresponding authors.
