# Supplementary material for: Evaluating the concept of three-dimensional printing guided endodontics in the dog
Source: Front Vet Sci. 2024 Nov 21;11:1481612. doi: 10.3389/fvets.2024.1481612 (PMC11617535; doi:10.3389/fvets.2024.1481612)
Supplement: Supplementary file 1 [file Table_1.DOCX]

Table S1. the diameter of the pulp crown of the mandible tooth

| Tooth number | Root position | Tooth crown/mm | Tooth number | Root position | Tooth crown/mm |
| --- | --- | --- | --- | --- | --- |
| 301 | / | 1.70 | 401 | / | 1.80 |
| 302 | / | 2.05 | 402 | / | 2.45 |
| 303 | / | 2.64 | 403 | / | 3.18 |
| 304 | / | 5.66 | 404 | / | 6.75 |
| 305 | / | 2.57 | 405 | / | 2.56 |
| 306 | mesial | 2.52 | 406 | mesial | 2.60 |
|  | distal | 3.10 |  | distal | 2.93 |
| 307 | mesial | 3.07 | 407 | mesial | 3.17 |
|  | distal | 3.58 |  | distal | 3.67 |
| 308 | mesial | 3.58 | 408 | mesial | 3.60 |
|  | distal | 3.81 |  | distal | 3.92 |
| 309 | mesial | 5.91 | 409 | mesial | 6.40 |
|  | distal | 5.05 |  | distal | 5.17 |
| 310 | mesial | 3.84 | 410 | mesial | 2.81 |
|  | distal | 4.01 |  | distal | 2.54 |

Table S2. The dimensions of beagle mandible teeth

| Tooth number | length/mm | width/mm | height/mm | Tooth number | length/mm | width/mm | height/mm |
| --- | --- | --- | --- | --- | --- | --- | --- |
| 301 | 3.16 | 3.93 | 6.63 | 401 | 3.60 | 3.89 | 7.52 |
| 302 | 3.22 | 3.98 | 7.62 | 402 | 4.61 | 4.85 | 7.30 |
| 303 | 4.14 | 5.08 | 6.83 | 403 | 4.49 | 5.38 | 8.24 |
| 304 | 5.44 | 7.55 | 17.75 | 404 | 5.31 | 8.34 | 17.44 |
| 305 | 4.80 | 3.87 | 5.12 | 405 | 4.39 | 3.14 | 5.97 |
| 306 | 8.82 | 4.32 | 8.97 | 406 | 8.75 | 4.19 | 8.67 |
| 307 | 10.45 | 5.89 | 9.11 | 407 | 10.41 | 5.96 | 9.03 |
| 308 | 11.81 | 6.67 | 9.02 | 408 | 11.44 | 6.21 | 8.54 |
| 309 | 20.43 | 7.87 | 13.95 | 409 | 19.61 | 7.45 | 13.80 |
| 310 | 9.31 | 6.80 | 5.30 | 410 | 9.53 | 7.20 | 5.66 |
